# *Lactobacillus fermentum* PC1 has the Capacity to Attenuate Joint Inflammation in Collagen-Induced Arthritis in DBA/1 Mice

**DOI:** 10.3390/nu11040785

**Published:** 2019-04-05

**Authors:** Meera Esvaran, Patricia L. Conway

**Affiliations:** 1 Centre for Marine Bio-Innovation, School of Biological, Earth and Environmental Sciences, The University of New South Wales, Sydney, NSW 2052, Australia; p.conway@unsw.edu.au; 2 NTU Food Technology Centre (NAFTEC), School of Chemical and Biomedical Engineering, Nanyang Technology University, 637459 Singapore, Singapore

**Keywords:** *L. fermentum* PC1, Collagen induced arthritis, inflammation

## Abstract

*Lactobacillus* strains have shown efficacy in attenuating inflammation. This study evaluated the potential of *Lactobacillus fermentum* PC1 for the treatment of rheumatoid arthritis (RA) using a murine model of collagen-induced arthritis. On Day 1, healthy DBA/1 mice (six to eight weeks of age) were immunized, with 100 μg of Chicken Type 11 collagen emulsified in complete Freund’s adjuvant (CFA) by intradermal injection, at the base of the tail. On Day 21, the mice were immunized intraperitoneally with 100 μg of Bovine Type11 collagen in phosphate buffered saline (PBS). On Day 28, the mice were immunized intraperitoneally with 50 μg of lipopolysaccharide (LPS). Viable *L. fermentum* PC1 (1 × 10^9^ colony forming units) was given daily from Day two until the end of the experiment. From Day 21 onwards, the mice were monitored daily for clinical signs of arthritis. On Day 44, the experiment was terminated. Paws were obtained for histology and serum for cytokine assays. *L. fermentum* PC1-fed mice had significantly reduced paw inflammation as well as decreased synovial infiltration and less cartilage damage. Circulating serum cytokine profiles revealed decreased IL-12 and increased anti-inflammatory cytokines, namely IL-4 and IL-10. Thus, early administration of *L.* *fermentum* PC1 could prove to be a valuable therapeutic agent in the management of RA.

## 1. Introduction

Rheumatoid arthritis (RA) is a widespread, potentially disabling disease which affects around 0.5–1.0% of the adult population worldwide. It is two to three times more prevalent in females than in males. It is a systemic disease that results in symmetrical joint inflammation along with constitutional symptoms, such as fatigue and depression. The symptoms of arthritis include pain, disability, and quality of life, which result in a considerable burden to the individual, health services, and society. The key to arthritis management is early diagnosis and treatment to prevent further joint destruction and maximize functional ability.

Collagen induced arthritis (CIA) in mice shares many similarities with human RA. As demonstrated in published reports, affected mice develop swollen joints [1,2], with a massive infiltration of inflammatory cells and subsequent cartilage damage in the joint synovium [3]. The earliest therapies for RA, such as non-steroid anti-inflammatory drugs (NSAIDs) treated the symptoms of RA. Unfortunately, NSAIDs have been shown to cause peptic and duodenal ulcers (reviewed in [4]). Currently there are other therapeutic drugs available that provide not just relief from symptoms, but also treat the disease (reviewed in [5,6]). However, these newer therapies are very expensive to manufacture and may have long term side-effects that are unknown.

The genus *Lactobacillus* belongs to the family of Lactobacteriaceae and has been used for centuries in bio-processing and preservation of food and feed. Studies using *Lactobacillus* species have demonstrated there is strain-dependent ability to confer immuno-modulatory promoting properties in a range of medical conditions in humans and animals. Strains of lactobacilli have been shown to reduce disease parameters in asthma [7], allergy [8], gastroenteritis [9], and inflammatory bowel disease [10]. Other researchers have found that feeding mice with either, *Lactobacillus delbrueckii* subsp. *bulgaricus* [11], or *Lactobacillus casei* [12], markedly inhibited the development of collagen-induced arthritis in mice. In the study by Kano et al. [11], the polysaccharides from one strain of *Lactobacillus bulgaricus* were found to prevent the onset of arthritis, while the bacterial cell walls of other strains, were not effective. However, there have also been reports of the cell wall of some strains of *Lactobacillus casei*, inducing arthritis in susceptible mice [13]. These studies demonstrate the efficacy of, and highlight the importance of, strain selection of *Lactobacillus* as therapeutic agents for a range of conditions

Previously, we have shown that *L. fermentum* PC1 can be used as an oral adjuvant [14]. The adjuvanticity was dependent on factors such as, the dosage of the antigen used, the nature of the antigen, and host genetics. Therefore, in this study we investigate if *L. fermentum* PC1 has the potential to confer protection against CIA to mice.

## 2. Materials and Methods 

### 2.1. Chemicals

Unless stated otherwise all chemicals were obtained from Sigma-Aldrich (Castle Hill, NSW, Australia).

### 2.2. Bacterial Strains and Growth Conditions

The lactobacillus strains, *Lactobacillus fermentum* FII 511400 (PC1), *Lactobacillus fermentum* FII 548700 (PC2), *Lactobacillus casei* FII 530500 (L26), *Lactobacillus casei* FII 540400 (L10), *Lactobacillus bulgaricus* FII 046900, and *Lactobacillus acidophilus* FII 10618 (LA) were obtained from the culture collection in the School of Biotechnology and Biomolecular Sciences, the University of New South Wales. All bacterial stock cultures were stored at −70 °C in 30% glycerol. *Lactobacillus* strains were grown in de Mann Rogosa Sharpe (MRS) broth (Thermo Fisher Scientific, Scoresby, Victoria, Australia) anaerobically (Don Whitley Scientific Mark 3 Anaerobic Chamber) at 37 °C. Glycerol stocks of lactobacilli were inoculated (1%) into MRS broth and propagated for 18h prior to use.

### 2.3. Cell Wall Preparations of Bacteria

Stationary-phase bacterial cultures (18 h) were centrifuged at 4000× *g* for 10 min. The resulting pellet was resuspended in 10ml of phosphate buffered saline (PBS; pH 7.2) and heat treated (80 °C; 30 min) to inactivate autolytic enzymes. The suspension was vortexed vigorously for 10 min. Cell walls were collected by centrifugation at 40,000× *g*, 4 °C, for 20 min. To remove nucleic acids, crude cell walls were first treated with DNase1and RNaseA (250 µg/mL), and then with trypsin (250 µg/mL) prior to being washed twice with PBS and once with distilled water between the steps. To remove the cell wall-associated proteins, the preparations were treated with papain (20 µg/mg). Purified cell walls were resuspended in PBS, and the suspensions were analyzed for protein content and sonicated for 20 min in an ice bath (Branson Ultrasonics, Danbury, CT, USA). The suspensions were centrifuged at 10,000× *g*, 4 °C, for 30 min and the supernatant collected for lysozyme assays.

### 2.4. Degradation of Cell Walls by Lysozyme

The cell wall preparations were resuspended in 0.1 M Na-acetate buffer (pH 5.0), to yield a concentration of 4 mg/mL, and incubated with 400 µg of lysozyme per ml for 24 h at 37 °C, mixing constantly on a platform shaker. The suspensions without lysozyme addition were used as controls. To calculate cell wall resistance to lysozyme, the decrease in the absorbance at 560 nm was measured and expressed as a percentage of the initial absorbance. 

### 2.5. Mice

Specific pathogen-free male DBA/1 mice, six to eight weeks old, obtained from the Animal Research Centre (Perth, Australia) were used for breeding. Mice used in experiments were six- to eight-week-old DBA/ 1 progeny mice bred and housed in the school animal facility in plastic cages (12 mice per cage) and fed *ad libitum* a commercial diet (Gordon’s Specialty Stockfeed, NSW, Australia) and autoclaved water. All animal experiments were approved by the UNSW animal ethics committee (UNSW ethics number: 03/28).

### 2.6. Induction of Collagen Induced Arthritis

Induction of arthritis was achieved using a well-established method [1]. Collagen from chicken sternal cartilage, Type 11 was dissolved in acetic acid to a concentration of 2 mg/mL by stirring overnight at 4 °C. On Day 1, male DBA/1 mice (8–12 weeks old) were immunized with 100 μg of Chicken Type 11 collagen emulsified in complete Freund’s adjuvant (CFA) by intradermal injection at the base of the tail. On Day 21, the mice were immunized intraperitoneally with 100 μg of Bovine Type 11 collagen in phosphate buffered saline (PBS). On Day 28, the mice were immunized intraperitoneally with 50μg of lipopolysaccharide (LPS) to synchronize onset of arthritis. Incidence of arthritis was 100%. The experiment was terminated on Day 44. Serum and paws of the mice were collected. 

### 2.7. Treatment with Lactobacillus fermentum PC1

Dosing of mice with viable *L. fermentum* PC1 commenced on Day 2 and continued until end of experiment. Each mouse was orally administered with 1 × 10^9^ colony forming units (CFU) of PC1 daily. Control mice were given an equal amount of PBS daily.

### 2.8. Assessment of Arthritis

Once the mice received the first immunization, they were monitored every third day. From Day 21 of the experiment, mice were monitored daily for clinical signs of arthritis. The arthritic score was graded as follows: 0) normal; 1) slight swelling and minimal erythema; 2) mild swelling and moderate erythema; 3) severe swelling and marked erythema. Paw swelling was assessed by measuring the thickness of the affected hind paws with 0–10-mm calipers. Each limb was graded, resulting in a maximal clinical score of 12 per animal. Once swelling was observed the mice were given Buprenorphine (0.1 mg/kg) as needed to ensure they remained pain-free.

### 2.9. Histological Analysis

Hind paws of the mice were removed post mortem at the end of the experiment. They were preserved in 10% formalin and then decalcified for 21 days in 10% EDTA followed by dehydration in increasing percentages of ethanol. These tissues were then cleared in xylene for 3h and embedded in paraffin blocks. The embedded organs were sectioned using microtome and stained with two stains; toluidine blue and hematoxylin and eosin to study the histopathological changes associated with the collagen induced arthritis. A person with no knowledge of the groupings, but considerable experience with RA research scored the slides using the following scoring system: 0) Normal; 1) Mild - some exudate in joint space or mild synovial changes, e.g., thickening, but cartilage and bone normal; 2) Moderate - exudative synovitis evident in >1 area, early pannus formation, cartilage generally OK or patchy change only; 3) Moderately severe - changes between grade 2 and 4; 4) Severe - widespread exudative synovitis with pannus and cartilage changes (e.g., aggrecan loss on toluidine blue stain) and some bone erosion; 5) Very severe - joint destruction or ankylosis with thick synovitis. For each mouse, one slide containing the two hind paws was scored.

### 2.10. Cytokine Assays

Cytokines IL-4, IL-10 and IL-12 were measured in serum using enzyme-linked immunosorbent assays (ELISA; Pharmingen, San Diego, CA, USA) according to the manufacturers’ instructions. 

### 2.11. Statistical Analysis

GraphPad Prism version 7.04 was used for data analysis. The results of the arthritis experiment are expressed as a mean ± SEM of 12 mice per group. The results were analyzed by two-tailed unpaired Students’ t-test with Welch’s correction. Data were considered statistically significant if *p* < 0.05.

## 3. Results

### 3.1. Arthogenicity of Bacterial Cell Walls and Safety of Lactobacilli 

Cell walls of some bacteria have been shown to induce arthritis in susceptible mice. All arthrogenic strains of bacteria have the same variation in the peptidoglycan (PG) layer. They have lysine as the third amino acid of the PG stem peptide in contrast to ornithine in the non-arthrogenic variation [1]. Previously, Smelyte et al. [2] demonstrated that the cell walls of arthrogenic bacteria are resistant to lysozyme. Therefore, we tested a panel of *Lactobacillus* strains for lysozyme sensitivity. The *L. casei* and *L. paracasei* were found to be lysozyme resistant. All other bacteria, including *L. fermentum* PC1, were found to be sensitive and thereby deemed safe to use (Table 1).

### 3.2. Arthritic Score

**Figure 1:**
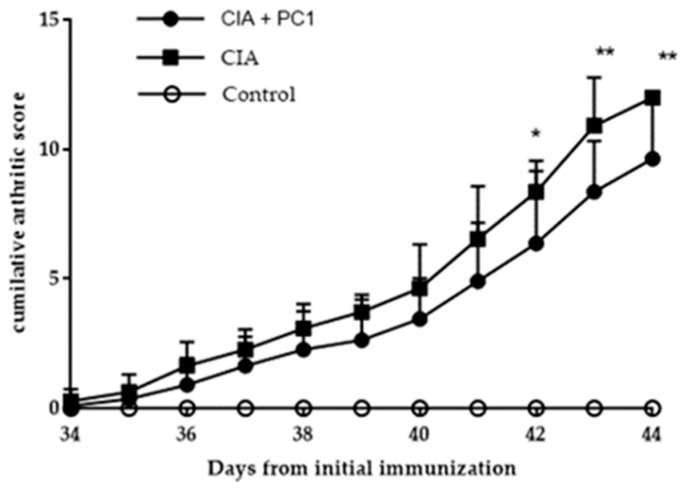
Arthritic scores in DBA/1 mice induced with collagen. Collagen-induced arthritis (CIA) mice were orally administered PBS daily from Day 2 until end of experiment. CIA + PC1 received viable *L. fermentum* PC1 (1 × 10^9^ colony forming units (CFU)) daily. Control were healthy age matched mice. Control mice were not induced with arthritis but received phosphate buffered saline (PBS). Arthritic scores were calculated as outlined in Materials and Methods. Results are expressed as the mean of 12 mice per group. Significant differences were indicated by * *p* < 0.05 and ** *p* < 0.01 as compared to CIA group.

All four paws of each mouse were examined and scored individually. The cumulative score of each mouse was calculated using the individual paw scores. Clinical symptoms started appearing in the mice by Day 34 after initial immunization with collagen. Inflammation and redness in the joints of mice increased steady as the study progressed. As shown in Figure 1, paw swelling was observed in both the control and PC1 treated mice. However, it was noted that the mice orally administered with PC1 (CIA + PC1) had less paw inflammation than the CIA mice who were given PBS. By Day 43, all CIA mice had swelling in all four paws, but in the PC1 treated group, the swelling of the paws was less pronounced. A few of the mice had swelling of all paws but the degree of swelling was less than in the CIA mice. On Day 42 the mice given PC1 had significantly less inflammation than the control (*p* = 0.04). On Days 43 and 44 of the experiment the significantly was *p* = 0.005 (Figure 1).

### 3.3. Histological Assessment of Arthritis

**Figure 2:**
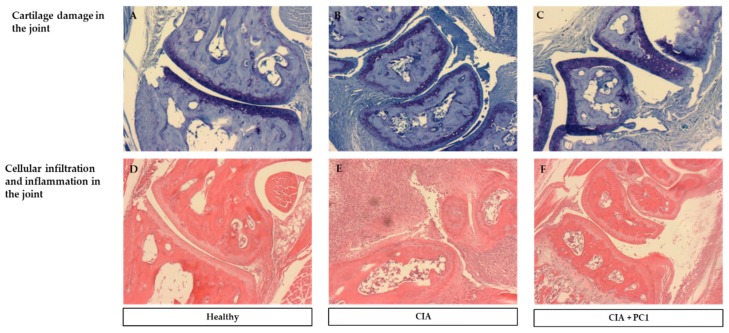
Representative histological staining with toluidine blue (×20) of paw joints of mice with collagen induced arthritis (CIA). (**A**) Healthy control mice show thick staining of the cartilage demonstrating lack of cartilage damage. (**B**) CIA mice have significantly less staining demonstrating breakdown of cartilage. (**C**) CIA mice given PC1 have less staining than the healthy mice but more than CIA mice indicating less cartilage damage. Hematoxylin and eosin staining of (**D**) healthy mice demonstrated no cellular infiltration. (**E**) CIA mice had massive cellular infiltration. (**F**) CIA mice given PC1 exhibited significantly less cellular infiltration than the CIA mice.

**Figure 3:**
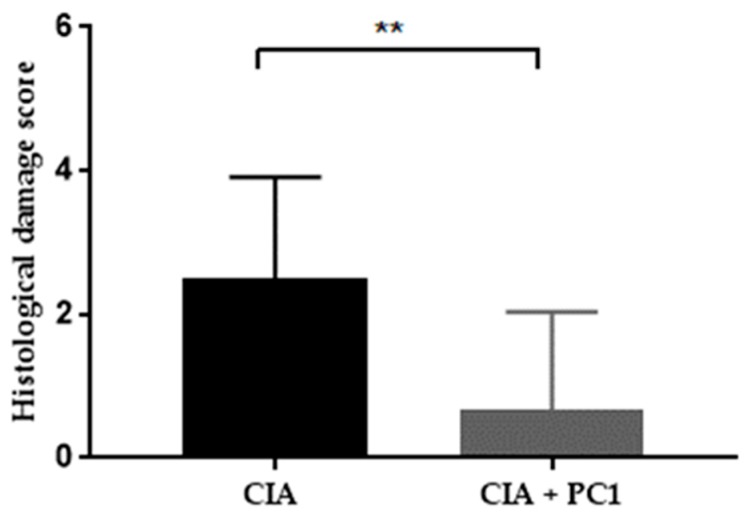
Effect of *L. fermentum* PC1 on histopathology in the joints of collagen induced arthritis (CIA) mice. The histological damage was scored as outlined in Materials and methods. Significant differences were indicated by ** *p* < 0.01 compared to CIA group. Each mouse was scored based on one slide with two hind paws in a blinded manner. Results are expressed as the average score per mouse (*n* = 12).

Histological analysis was performed on both hind legs of each mouse at the termination of the experiment. As shown in Figure 2, control mice (A and D) displayed no histological signs of arthritis. They had no inflammation or cellular infiltration (D) and no cartilage damage, as noted by the thick continuous straining of toluidine blue (A). The mice with CIA (B and E), as well as those treated with PC1 (C and F), demonstrated pathological signs of arthritis. The CIA mice revealed massive infiltration of inflammatory cells (E). They also exhibited substantial cartilage damage as evidenced by the very thin layer of toluidine blue (B). However, the mice treated with PC1 had significantly less inflammatory cell infiltration (F) and cartilage damage (C) in the joints than was seen in (*p* = 0.0097) the CIA mice (Figure 3). It was noted that all mice with CIA exhibited cartilage damage, whereas not all the mice treated with PC1 had cartilage damage. The average histopathological damage in the groups is shown in Figure 3. 

### 3.4. Effect of L. fermentum PC1 on Systemic Cytokine Levels

**Figure 4:**
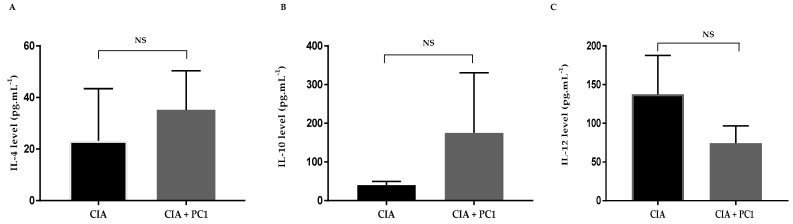
Levels of cytokines IL-4 (**A**), IL-10 (**B**) and IL-12 (**C**) after *L. fermentum* PC1 administration in collagen induced arthritis (CIA) mice. CIA mice were orally administered PBS daily from Day 2 until end of experiment. CIA + PC1 received viable *L. fermentum* PC1 (1 × 10^9^ CFU) daily. Control were healthy age matched mice. Serum levels of cytokines IL4, IL-10 and IL-12 were measured by ELISA. Results are shown as the mean ± SEM of 12 mice per group. NS denotes non-significance between the groups tested.

In order to examine whether the administration of PC1 could induce a shift from Th1 to a more Th2 immune responses, we determined cytokine responses in serum. Serum was collected at the termination of the experiment to examine systemic immune responses. The level of both anti-inflammatory cytokines, IL-4 and IL-10 were increased by treatment of PC1. Cytokine IL-4 increased from 23.27 ± 11.64 in the CIA group to 35.24 ± 7.57 in CIA + PC1 group (*p* = 0.407). Cytokine IL-10 also increased from 134.90 ± 89.52 in the CIA group to 175.6 0 ± 89.41 in CIA + PC1 group (*p* = 0.132). In contrast, administration of PC1 resulted in a decrease in the level of pro-inflammatory cytokine IL-12 from 137.70 ± 25.03 in the CIA to 74.66 ± 10.94 in CIA + PC1 group (*p* = 0.06). As noted, the differences in cytokines between the groups were not statistically different (Figure 4).

## 4. Discussion

We have previously shown that *L. fermentum* PC1 has immune-adjuvant properties in mice [3]. The aim of the present study was to explore the potential of *L. fermentum* PC1, as a therapeutic agent in rheumatoid arthritis, using a murine model of collagen induced arthritis. 

There are numerous studies showing the efficacy of lactobacilli strains in ameliorating CIA [4,5,6,7,8,9] in rodents. However, there are also reports demonstrating that the cell wall of bacteria, including lactobacilli strains can induce arthritis in susceptible [2,10] hosts. It has been shown that the cell wall of bacteria resistant to lysozyme can be arthrogenic [11,12]. Therefore, we first tested a panel of lactobacillus strains for their resistance to lysozyme. *L. fermentum* PC1 was found to be sensitive to lysozyme. Therefore, PC1 was deemed to be non-arthritogenic and safe to use in the CIA murine model. 

In our study, daily dosing with viable *L. fermentum* PC1 exerted a protective effect on collagen-induced arthritis symptoms in mice. This was demonstrated visually by decreased paw inflammation. Histological analysis revealed less cellular infiltration and reduced cartilage damage in the paw joints at the termination of the experiment. Circulating serum cytokine analyses demonstrated a decrease in pro-inflammatory cytokine IL-12 and increased anti-inflammatory cytokines, IL-4 and IL-10. Our results are consistent with other studies, using probiotics that have correlated protection against CIA with a decrease of pro-inflammatory cytokines [4,13,14]. 

The exact etiology of RA remains unknown, but one of the first visual signs of RA is paw-swelling, caused by inflammation in the affected joints. Joint inflammation is characterized by the increase in pro-inflammatory cytokines IL-1, TNF-α, IL-17, IL-18, and RANK ligand (RANKL) [15,16]. Cytokine IL-12 has been shown to be elevated in the serum and joints of CIA mice [17,18]. In the present study, elevated levels of serum IL-12 were noted in the CIA mice. However, when *L. fermentum* PC1 was administered daily after the immunization with collagen, IL-12 levels decreased significantly. Anti-inflammatory cytokines, IL-4 and IL-10 are integral in reducing arthritic symptoms in CIA mice. IL-10 has an opposing action to IL-12. It decreases the release of pro-inflammatory cytokines from macrophages and stimulates B cell function. It also promotes the development of a Th2 type cytokine pattern by inhibiting the IFN-γ production by T lymphocytes and NK cells, particularly via the suppression of IL-12 synthesis in accessory cells. Studies have shown the importance of IL-4 and IL-10 in the control of arthritis because blocking these cytokines has resulted in the accelerated onset of CIA [15,19]. Treatment with recombinant IL-4 antibody alone does not ameliorate arthritic symptoms, but when given in combination with recombinant IL-10 antibody, leads to reduced cellular infiltrations in synovial tissue [19]. Cytokine IL-4, together with IL-10, are believed to mediate regulatory effect by suppression of IFN-γ and IL-17 expression (reviewed in [15,20]). Increased IL-4 has also been linked to cartilage protection [20]. The present study does corroborate existing literature, as elevated IL-4 and IL-10 serum levels seem to correlate with less joint inflammation and cartilage damage. 

At present, it is not known what component of *L. fermentum* PC1 mediates protection. However, other workers have demonstrated the anti-inflammatory capacity of cell wall components [21] and soluble secreted molecules [22] by lactobacillus. Specifically, the exopolysaccharides of lactobacilli strains can confer protection against CIA [5,9]. 

Different lactobacilli strains mediate an anti-inflammatory effect by inhibiting different inflammatory pathways. The anti-inflammatory effect by lactobacilli fermented food, Oyaksungisan, is mediated via the inhibition of NF-κB pathway and MAPK activity [23]. Whereas, a soluble molecule from *Lactobacillus reuteri* CRL 1098 inhibited NF-κB and PI3K pathway [24]. *Lactobacillus casei* has been reported to mediate suppression of experimental arthritis by inhibiting the nuclear translocation of NF-κB [25]. Further work needs to be done to elucidate what component of *L. fermentum* PC1 is responsible for exerting the effects seen in this study. 

## 5. Conclusions

Our study demonstrates that the oral administration of viable *L. fermentum* PC1 daily, after immunizing with collagen, significantly decreased joint inflammation. This effect appears to be mediated by the increase in endogenous IL-4 and IL-10 cytokines, which suppressed IL-12 levels detected in the serum. Although the results are very promising, additional work needs to be done to establish the efficacy of *L. fermentum* PC1 as a therapeutic agent in the treatment of rheumatoid arthritis. 

## Figures and Tables

**Table 1:** Lysozyme sensitivity of cell walls was assayed by measuring the absorbance of bacterial cell wall suspension prior to incubation with lysozyme (4 mg/mL) for 24 h at 37 °C. The absorbance of the cell wall suspensions was measured again and expressed as a percentage of the initial absorbance.

Bacterial Strain	% Lysozyme Degradation
*L. paracasei* L26	3 ± 0.93
*L. casei* L10	0.29 ± 0.13
*L. bulgaricus*	52 ± 3.59
*L. fermentum* PC1	58 ± 3.87
*L. fermentum* PC2	50 ± 3.872
*L. acidophilus* LA	53 ± 4.25

## References

[1] Zhang X., Rimpilainen M., Simelyte E., Toivanen P. (2000). What determines arthritogenicity of bacterial cell wall? A study on Eubacterium cell wall-induced arthritis. Rheumatology (Oxford).

[2] Simelyte E., Rimpilainen M., Lehtonen L., Zhang X., Toivanen P. (2000). Bacterial cell wall-induced arthritis: Chemical composition and tissue distribution of four Lactobacillus strains. Infect. Immun..

[3] Esvaran M., Conway P.L. (2016). Factors that Influence the Immunological Adjuvant Effect of *Lactobacillus fermentum* PC1 on Specific Immune Responses in Mice to Orally Administered Antigens. Vaccines (Basel).

[4] Amdekar S., Singh V., Singh R., Sharma P., Keshav P., Kumar A. (2011). *Lactobacillus casei reduces* the inflammatory joint damage associated with collagen-induced arthritis (CIA) by reducing the pro-inflammatory cytokines: *Lactobacillus casei*: COX-2 inhibitor. J. Clin. Immunol..

[5] Nowak B., Ciszek-Lenda M., Srottek M., Gamian A., Kontny E., Gorska-Fraczek S., Marcinkiewicz J. (2012). *Lactobacillus rhamnosus* exopolysaccharide ameliorates arthritis induced by the systemic injection of collagen and lipopolysaccharide in DBA/1 mice. Arch. Immunol. Ther. Exp..

[6] Kano H., Kaneko T., Kaminogawa S. (2002). Oral intake of *Lactobacillus delbrueckii* subsp. *bulgaricus* OLL1073R-1 prevents collagen-induced arthritis in mice. J. Food Prot..

[7] Kato I., Endo-Tanaka K., Yokokura T. (1998). Suppressive effects of the oral administration of *Lactobacillus casei* on type II collagen-induced arthritis in DBA/1 mice. Life Sci..

[8] Liu X., Zhang J., Zou Q., Zhong B., Wang H., Mou F., Wu L., Fang Y. (2016). *Lactobacillus salivarius* Isolated from Patients with Rheumatoid Arthritis Suppresses Collagen-Induced Arthritis and Increases Treg Frequency in Mice. J. Interferon Cytokine Res..

[9] Sugihara R., Yoshimura M., Mori M., Kanayama N., Hikida M., Ohmori H. (2000). Prevention of collagen-induced arthritis in DBA/1 mice by oral administration of AZ-9, a bacterial polysaccharide from *Klebsiella oxytoca*. Immunopharmacology.

[10] Lehman T.J., Allen J.B., Plotz P.H., Wilder R.L. (1985). Bacterial cell wall composition, lysozyme resistance, and the induction of chronic arthritis in rats. Rheumatol. Int..

[11] Simelyte E., Rimpilainen M., Zhang X., Toivanen P. (2003). Role of peptidoglycan subtypes in the pathogenesis of bacterial cell wall arthritis. Ann. Rheum. Dis..

[12] Zhang X., Rimpilainen M., Hoffmann B., Simelyte E., Aho H., Toivanen P. (2001). Experimental chronic arthritis and granulomatous inflammation induced by bifidobacterium cell walls. Scand. J. Immunol..

[13] Kano H., Mogami O., Uchida M. (2002). Oral administration of milk fermented with *Lactobacillus delbrueckii* ssp. *bulgaricus* OLL1073R-1 to DBA/1 mice inhibits secretion of proinflammatory cytokines. Cytotechnology.

[14] So J.S., Song M.K., Kwon H.K., Lee C.G., Chae C.S., Sahoo A., Jash A., Lee S.H., Park Z.Y., Im S.H. (2011). *Lactobacillus casei* enhances type II collagen/glucosamine-mediated suppression of inflammatory responses in experimental osteoarthritis. Life Sci..

[15] Lubberts E., van den Berg W.B. (2003). Cytokines in the pathogenesis of rheumatoid arthritis and collagen-induced arthritis. Adv. Exp. Med. Biol..

[16] Brennan F.M., McInnes I.B. (2008). Evidence that cytokines play a role in rheumatoid arthritis. J. Clin. Investig..

[17] Zheng K., Chen Z., Sun W., Liu B., Fan D., Guo Q., Luo H., Shen J., Li L., He X. (2018). Hei-Gu-Teng Zhuifenghuoluo Granule Modulates IL-12 Signal Pathway to Inhibit the Inflammatory Response in Rheumatoid Arthritis. J. Immunol. Res..

[18] Liu W., Zhang Y., Zhu W., Ma C., Ruan J., Long H., Wang Y. (2018). Sinomenine Inhibits the Progression of Rheumatoid Arthritis by Regulating the Secretion of Inflammatory Cytokines and Monocyte/Macrophage Subsets. Front. Immunol..

[19] Joosten L., Lubberts E., Durez P., Helsen M., Jacobs M., Goldman Md M., Berg W. (1997). Role of interleukin-4 and interleukin-10 in murine collagen-induced arthritis. Protective effect of interleukin-4 and interleukin-10 treatment on cartilage destruction. Arthritis Rheum..

[20] Sarkar S., Cooney L.A., White P., Dunlop D.B., Endres J., Jorns J.M., Wasco M.J., Fox D.A. (2009). Regulation of pathogenic IL-17 responses in collagen-induced arthritis: Roles of endogenous interferon-gamma and IL-4. Arthritis Res. Ther..

[21] Rong J., Zheng H., Liu M., Hu X., Wang T., Zhang X., Jin F., Wang L. (2015). Probiotic and anti-inflammatory attributes of an isolate *Lactobacillus helveticus* NS8 from Mongolian fermented koumiss. BMC Microbiol..

[22] Bleau C., Monges A., Rashidan K., Laverdure J.P., Lacroix M., Van Calsteren M.R., Millette M., Savard R., Lamontagne L. (2010). Intermediate chains of exopolysaccharides from *Lactobacillus rhamnosus* RW-9595M increase IL-10 production by macrophages. J. Appl. Microbiol..

[23] Oh Y.C., Cho W.K., Oh J.H., Im G.Y., Jeong Y.H., Yang M.C., Ma J.Y. (2012). Fermentation by *Lactobacillus* enhances anti-inflammatory effect of Oyaksungisan on LPS-stimulated RAW 264.7 mouse macrophage cells. BMC Complement. Altern. Med..

[24] Griet M., Zelaya H., Mateos M.V., Salva S., Juarez G.E., de Valdez G.F., Villena J., Salvador G.A., Rodriguez A.V. (2014). Soluble factors from *Lactobacillus reuteri* CRL1098 have anti-inflammatory effects in acute lung injury induced by lipopolysaccharide in mice. PLoS ONE.

[25] So J.S., Kwon H.K., Lee C.G., Yi H.J., Park J.A., Lim S.Y., Hwang K.C., Jeon Y.H., Im S.H. (2008). *Lactobacillus casei* suppresses experimental arthritis by down-regulating T helper 1 effector functions. Mol. Immunol..

